# Molecular Basis of the Function of Transcriptional Enhancers

**DOI:** 10.3390/cells9071620

**Published:** 2020-07-05

**Authors:** Airat N. Ibragimov, Oleg V. Bylino, Yulii V. Shidlovskii

**Affiliations:** 1 Laboratory of Gene Expression Regulation in Development, Institute of Gene Biology, Russian Academy of Sciences, 34/5 Vavilov St., 119334 Moscow, Russia; dysport@bk.ru (A.N.I.); bylino@gmail.com (O.V.B.); 2 Center for Precision Genome Editing and Genetic Technologies for Biomedicine, Institute of Gene Biology, Russian Academy of Sciences, 34/5 Vavilov St., 119334 Moscow, Russia; 3 I.M. Sechenov First Moscow State Medical University, 8, bldg. 2 Trubetskaya St., 119048 Moscow, Russia

**Keywords:** enhancer, promoter, chromatin, transcriptional bursting, transcription factories, enhancer RNA, epigenetic marks

## Abstract

Transcriptional enhancers are major genomic elements that control gene activity in eukaryotes. Recent studies provided deeper insight into the temporal and spatial organization of transcription in the nucleus, the role of non-coding RNAs in the process, and the epigenetic control of gene expression. Thus, multiple molecular details of enhancer functioning were revealed. Here, we describe the recent data and models of molecular organization of enhancer-driven transcription.

## 1. Introduction

Gene transcription is precisely organized in time and space. The process requires the participation of hundreds of molecules, which form an extensive interaction network. Substantial progress was achieved recently in our understanding of the molecular processes that take place in the cell nucleus (e.g., see [1,2,3,4,5,6,7,8,9]). Novel techniques revealed multiple unexpected phenomena occurring in chromatin. Here, we discuss the novel ideas and models that appeared in the field of molecular organization of enhancer functioning.

## 2. Temporal Organization of Enhancer-Dependent Transcription. Transcriptional Bursting

**Figure 1:**
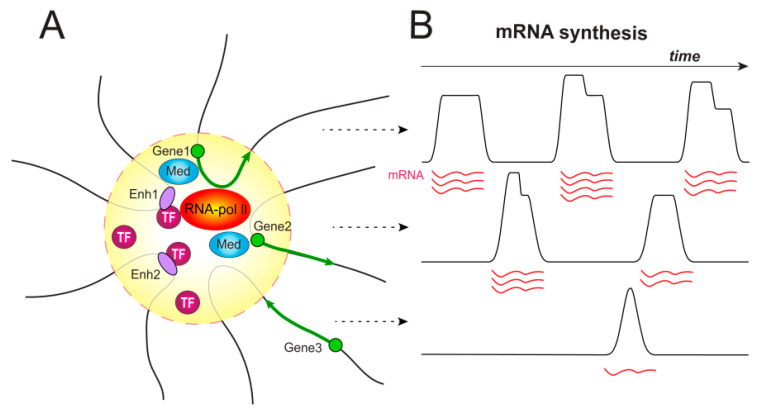
Transcription factory and transcriptional bursting. (**A**) Enhancers and promoters of several genes located in different chromosomal positions or even on different chromosomes cluster together in a focus within the cell nucleus during transcription [19,20,21]. These discrete nuclear foci, which contain the phosphorylated form of RNA polymerase II, are called transcription factories. A transcription factory represents a phase-separated droplet, where the concentration of proteins can be 100 times higher than in the surrounding nucleoplasm [22,23,24]. The high concentration of transcription factors in the transcription factory promotes additional rounds of transcription initiation, facilitating the interaction between regulatory elements. Three potentially co-expressed genes share one transcription factory. Chromatin fibers are decondensed (thin gray line) inside the factory and condensed outside (thick black line). TF, transcription factor; Med, the Mediator complex; Enh, enhancer; yellow circle, transcription factory. (**B**) A hypothetical vision of the organization of bursting in space in eukaryotes is represented. Gene1 is expressed and located within a transcription factory at a given time point. Gene2 has already been expressed and leaves the transcription factory now. Gene3 now returns to the factory. In accordance with the model described in [25], the factory includes only one polymerase molecule, thus producing non-overlapping peaks of mRNA. The regulation of transcription in one factory can be carried out by multiple enhancers (only two enhancers are shown).

Eukaryotic mRNA synthesis proceeds in short intense pulses, or bursts, which are periods of gene transcriptional activity followed by periods of gene silence. The intermittent pulsation of gene expression in eukaryotes is an intrinsic property of eukaryotic transcription and chromatin organization and is not caused by any external factor [10]. Bursts are characterized using several interrelated parameters, such as the peak amplitude (peak height, intensity), peak duration (time, length), and frequency of bursts (rate of re-occurrence, burst fraction, the number of bursts). The amplitude and duration taken together are sometimes called the burst size and contrasted with the burst frequency. All three parameters affect the transcription output, which is expressed in the amount of synthesized mRNA of the gene (Figure 1). The peak duration and frequency are the main burst characteristics, while the amplitude reflects the burst kinetics and can vary during one burst [11]. The transcriptional kinetics is highly gene specific and markedly altered by *cis*-regulatory DNA elements [12]. The average burst time was calculated for some organisms and is of the order of 5 min [13]. 

As a phenomenon, transcriptional bursting might represent a source of intrinsically random fluctuations in mRNA and protein levels in the cell. Bursts in mRNA level may be compensated for by decreases in the degradation levels of the respective proteins [14]. An increase in gene expression may result not only from changes in bursting parameters, but also from an increase in the number of cells expressing the gene in a cell population. The proportion of cells expressing a definite gene was found to greatly vary during different stages of development [13].

The level of gene activity depends on the frequency of transcriptional bursts [11]. Highly expressed genes are characterized by long bursts interrupted by short breaks, while low-expressed genes are characterized by shorter bursts and longer breaks [15]. Enhancers regulate the frequency of transcriptional bursts [11,16]. Different developmental enhancers produce transcriptional bursts of the reporter gene with similar amplitudes and duration, but they generate very different bursting frequencies, strong enhancers producing more bursts than weak enhancers [11]. The insertion of an insulator reduces the frequency of bursts and the corresponding level of gene expression, but not co-activation of the linked genes, suggesting that enhancer regulation of the bursting frequency is a key parameter of gene control [11]. However, the bursting frequency does not change in response to an increase in the number of binding sites for a transcriptional activator on an enhancer, while cells with an increased burst size were found to appear in a cell culture in this case [14]. 

Transcriptional bursts coincide with promoter–enhancer chromatin contacts in tissues, indicating that enhancer–promoter chromatin looping acts as a regulatory mechanism [16]. In early observations, it was noticed that the length and height of a pulse are the same at different stages of development, although the frequency of pulses may vary slightly [13]. However, later studies using the Hbb globin locus as a model showed that targeted enhancer–promoter looping increases the frequency of bursting, but not the burst duration [17]. The discrepancy between the earlier and later data can be explained by different models chosen for research. To increase both the frequency and the amplitude (intensity) of bursts, a second event was found to be necessary in addition to the spatial convergence of the two regulatory elements. The induction of cell differentiation is this event in the case of LCR of beta-globin genes [17], being likely accompanied by the recruitment of additional activators or chromatin modifying complexes to the enhancer. 

In cells where two developmentally distinct LCR-regulated globin genes are co-transcribed *in cis*, the burst sizes, but not burst frequencies, of both genes were comparable. Furthermore, the co-transcription of both genes was statistically mutually inhibitory [17]. Thus, promoter competition for one enhancer *in cis* accounts for the alternation in burst frequency, emphasizing that changes in enhancer–promoter contacts do not alter the burst size, but affect only the burst frequency. The finding supports the model that the enhancer rapidly switches between promoters to allow co-transcriptional events to occur, suggesting that enhancer–promoter loops are formed and released with rapid kinetics. At the special level, such genes can be located in different transcription factories (see below) or be in the same factory. Definitive evidence for this model would require a direct imaging of enhancer–promoter contacts in live cells. 

How does chromatin control the bursting events? At the very beginning of bursting research, re-expression was shown to be more likely to arise than de novo transcription in a cell population, indicating that epigenetic processes and transcription memory are involved in controlling gene expression [13]. It was proposed that random chromatin remodeling events can be sources of bursting [14]. Indeed, it was found subsequently that the state of chromatin affects all bursting parameters (for a review, see [10]).

How may bursting be specially organized? It was noted that when two genes are integrated close together into the genome, they exhibit the same bursts [14], suggesting that two or more genes might be co-regulated by one enhancer in one place of the cell nucleus. It was later confirmed that a single enhancer can co-activate two reporter genes linked with the enhancer, the two genes exhibiting coordinated bursting profiles when regulated by such as a shared enhancer [11]. The finding emphasizes the importance of the chromosome topology in gene control by shared enhancers. It was demonstrated recently that upon transvection, two alleles regulated by the same enhancer very weakly compete with each other and are spatially co-expressed, reinforcing the possibility that they share a common pool of transcription machinery [18].

Thus, eukaryotic transcription occurs in short bursts. Enhancers regulate the total gene expression output predominately by modulating the bursting frequency.

## 3. Spatial Organization of Enhancer-Dependent Transcription in the Nuclear Space

### 3.1. Transcription Factories

According to the current views of how transcription is organized in eukaryotes, the enhancers and promoters of co-expressed genes located in different chromosomal positions or even on different chromosomes cluster together during transcription to produce discrete nuclear foci, which contain the phosphorylated RNA polymerase II (Pol II) form and are known as transcription factories [20,26,27,28,29] (Figure 1). Since the factories observed in a cell are fewer than the genes transcribed at the given time point and because the factory formation time is approximately 5 s in normal conditions and increases by one order of magnitude (50 s) when expression is induced by a potent factor (serum), the process is now understood as transient, highly dynamic, and strongly depending on the identity of the genes whose regions are in contact with a factory [30]. Clusters increase in size upon strong stimulation, but the inhibition of Pol II elongation does not prevent the formation of transcription factories, indicating that they form at the initiation stage [30,31]. Moreover, elongation and initiation seem to occur in different compartments. Initiation takes place within a factory, while elongation occurs outside, in the space immediately adjacent to the factory [32]. When fluorescently labeled molecules of Pol II are used, fluorescence decreases after initiation because the molecules start elongation and possibly cease to cluster together. Live imaging studies showed recently that the mobility of regulatory elements, especially enhancers, increased with transcriptional activation upon the differentiation of embryonic stem cells (ESCs) [33]. The increase most likely correlates with their recruitment and the de novo formation of a transcription factory. 

### 3.2. Phase Separation

Recent findings shed some light on why many transcription factors (TFs) and co-activators contain the so-called low-complexity domains (LCDs), which are regions with low amino acid diversity and are also known as the intrinsically disordered regions. LCDs are capable of forming liquid-like condensates, which are possible to disrupt using chemicals that perturb condensates, for example, with hexanediols [24]. Proteins with LCDs form phase-separated droplets, which help to compartmentalize and concentrate the transcription apparatus components and facilitate the liquid–liquid phase transitions [34,35,36]. The protein concentration in phase-separated droplets is 100 times that in the surrounding liquid phase [37]. It was shown that droplets formed by the C-terminal Pol II domain may include intact Pol II and are disrupted upon the phosphorylation of transcription initiation factor IIH kinase CDK7 [22]. As for TFs, their activation domains are capable of forming phase-separated droplets with Mediator in vitro, and their activation in vivo depends on the same amino acid residues [38]. Thus, synchronous cycles of gene recruitment to transcription factories dynamically arising de novo may explain transcriptional bursting; in turn, transcriptional bursting is facilitated by phase separation. Pre-assembled gene topologies may serve as hubs or traps in this case to accumulate Pol II and other complexes required for gene expression. A higher concentration of trans-acting factors in phase-separated droplets will promote the re-initiation cycles, increasing the likelihood of stochastic encounters between specific combinations of regulatory elements that would otherwise occur only very rarely in the nucleus and creating the microenvironment to boost gene expression [39,40]. All of these processes are accompanied by intense movements of regulatory elements and especially enhancers [33].

As for enhancer-dependent activation, the above data suggest that activation must be associated with a phase switching, when one of the regulatory elements, for example, the gene promoter, is recruited from one—the liquid phase—to another, the protein phase, where the enhancer resides. Once the elements are in the same phase enriched in TFs, Mediator, Pol II, etc., their higher concentrations may facilitate the Linking/Chaining/Looping between the regulatory elements. A physical contact between the enhancer and promoter is not essential for transcriptional activation; it is enough that they are brought to a distance shorter than 330 nm of each other [18]. On the other hand, the Pol II concentration may not be as high as assumed in transcription factory assembly sites at the corresponding phase because data obtained using reflected light-sheet super-resolution microscopy indicate that more than 70% of the transcription sites contain only one Pol II molecule, contradicting the existing models [25].

### 3.3. Does a Polymerase Move along a Gene or Does a Gene Move through a Polymerase?

**Figure 2:**
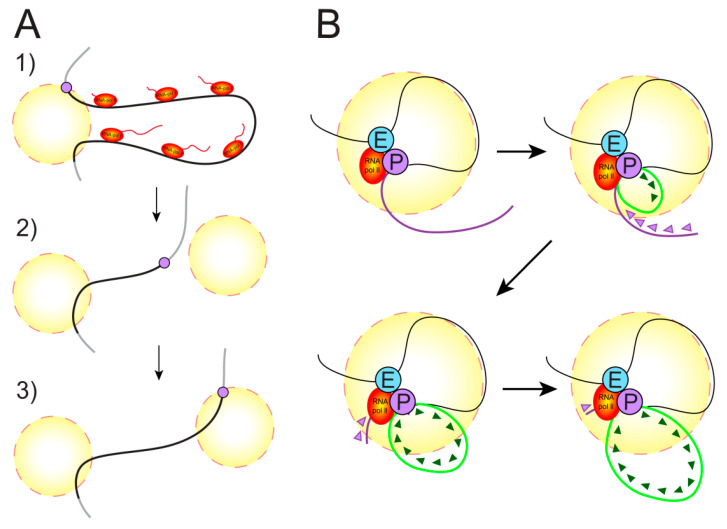
Spatial organization of the gene body within a transcription factory (yellow circle). (**A**) The organization of a factory for extended genes is presented. Genes occurring in such a factory form the so-called gene loops, when the beginning of the gene and its end somehow become anchored in the factory [15]. In this case, the gene looks similar to a loop of chromatin. Pol II molecules move along the gene one after another, reading the gene. A gene can be disconnected from the factory, and re-initiation can occur in another factory, where the tail of the gene is subsequently pulled [7,42]. (**B**) An alternative form of transcription organization for extended genes is presented. The gene enhancer and promoter are brought together and possibly are in a complex. Moreover, part of the gene is located inside the factory, and part of the gene is located on the surface of the factory or is partly reeled on it [41]. The Pol II molecule is immobilized in the factory, and the gene body is pulled through it in the form of a loop formed [43]. The spatial organization of transcription for short genes in a transcription factory was not studied because of technical limitations and may proceed according to either of the two presented scenarios [43].

The above data clarify, to an extent, the nature of enhancer function and the organization of eukaryotic transcription. Yet the question is still open as to whether Pol II moves along the gene during transcription or the gene moves relative to Pol II immobilized in a dynamically forming factory. Studies with 3C methods and super-resolution microscopy showed that the distance between two transcripts of two co-expressed genes co-localized in one transcription factory is lying within 35 nm of the surface of an 87-nm sphere. Given the known gene sizes, the finding is only possible to explain by assuming that two responding genes diffuse to an 87-nm factory to be transcribed by immobilized enzymes and that when two genes are reeled in to the transcription factory, only parts being transcribed at a given moment lie transiently together [41]. The same research team visualized the process of induced transcription on the template of a long gene (221 kb). It was found that the gene promoter is initially bound in a transcription factory and that the template is then drawn through polymerase immobilized in the factory. According to the model assumed, the promoter remains in the vicinity of the factory for a while, but it then moves away and loses its association with the factory so that re-initiation may take place in another factory [7,42]. As shown more recently with synchronized cells, a contact of the enhancer with the promoter is preserved during the transcription of a gene. However, the polymerase remains immobilized in the factory, and the DNA-coding region of the gene extends through Pol II outside the factory in the form of an ever-increasing loop [43] (Figure 2).

Recently, evidence was provided in favor of an alternative point of view. Using fluorescence in situ hybridization (FISH) on very long genes, it was demonstrated that such genes form transcriptional loops and that the bases of these loops are not brought together. It was found that loops extend into the nuclear interior where euchromatin is situated and are decorated with ribonucleoprotein complexes containing moving Pol II [15] (Figure 2).

Thus, different scenarios are possible for different genes in different conditions. The enhancer–polymerase complex may move along a chromatin fiber during transcription elongation or, oppositely, it may lie within a transcription factory while the gene body is drawn through the factory and looped out to gradually move away. 

### 3.4. Enhancer–Promoter Contacts and Transcription Initiation

#### 3.4.1. Two Mechanisms of Eukaryotic Transcription Initiation.

The mechanisms of enhancer-dependent activation of gene transcription are tightly associated with how Pol II works on the given gene. Two basic models currently explain the activation of gene transcription in higher eukaryotes. One model assumes that Pol II is absent from the promoter and coding region when a gene is repressed (inactive). An activation signal—for example, a hormonal or stress stimulus—serves to recruit a transcriptional activator to the promoter and/or enhancer of the gene. The activator stimulates the recruitment of Pol II and assembly of the Pol II complex. Once bound to the promoter, Pol II immediately starts elongation on the coding region and departs after having completed transcription until the next recruitment round. Recruitment of the Pol II complex is a limiting step of the process in this case. 

The other mechanism is assumed for the genes where the Pol II complex is efficiently recruited to the promoter even when the gene is inactive. The complex pre-assembled on the promoter of an inactive gene undergoes all of the transcription initiation steps. The C-terminal domain of Pol II is phosphorylated at Ser5 by Cdk7 kinase, and the complex leaves the promoter to start RNA synthesis. After adding 40–60 nucleotides to RNA, the Pol II complex is stalled by the negative elongation factor (NELF, which consists of WHSC2/NELF A, COBRA1/NELF B, THIL/NELF C/D, and NELF E subunits) and DRB sensitivity inducing factor (DSIF, which consists of SPT5/SPT4 subunits) and waits for a signal to start productive transcription. An activator recruited to the promoter facilitates the transition of the promoter-associated complex into the elongating state. Elongation kinase positive transcription elongation factor (P-TEFb, which consists of CycT and Cdk9) phosphorylates the subunits NELF-A and NELF-E of NELF and the SPT5 subunit of DSIF, as well as Ser2 in C-terminal domain of Pol II [44,45]. NELF leaves the Pol II complex, while phosphorylated DSIF now acts as a positive regulator of Pol II, and the enzyme continues moving. Translocation of the Pol II complex from the promoter to the coding gene region is a limiting step of this mechanism. The Pol II abundance does not change on the promoters of such genes after their transcriptional activation, but it significantly increases in the coding regions [46]. 

#### 3.4.2. Two Types of Eukaryotic Pol II Promoters Differing in Initiation Mode

Thus, two types of promoters are recognized: the promoters that are associated with paused Pol II and are regulated by stalling the Pol II complex (the inhibition phenomenon, or distorted Pol II elongation) and the stochastic promoters, which are characterized by Pol II complex turnover. A substantial proportion of active genes was found to have stalled Pol II on the promoter in *Drosophila* [47]. The mechanism that regulates transcription by stalling Pol II is mostly characteristic of developmental and signal transduction genes and is almost not observed in the case of housekeeping genes [48]. The promoters and coding regions differ in nucleosome abundance between the two gene groups, i.e., highly regulated genes have nucleosome-rich promoters and rare nucleosomes in the gene body, while housekeeping genes have nucleosome-depleted promoters and a high nucleosome frequency in the coding regions [49]. The enhancer-dependent expression regulation may be associated with the different modes of Pol II function on promoters of the two basically different types. For example, enhancers may act to stimulate the Pol II transition from the paused state to elongation rather than Pol II recruitment and assembly of the preinitiation complex on the promoters of highly regulated developmental genes. 

#### 3.4.3. Two Basic Models of Enhancer Action Associated with Transcription Initiation and the Problem of Proximity

In accordance with the above mechanisms, two basic models were assumed for the mechanism of action of enhancers. One is a permissive model and suggests that enhancer–promoter contacts pre-exist before gene activation (e.g., in precursor cells) and are only slightly reorganized in the course of cell differentiation (specification), as was demonstrated for TNF-alpha-, thrombopoietin-, and p53-dependent genes [50,51,52]. In all of these cases, a pre-existing chromatin topology is used to maximally reduce the search area and time for establishing enhancer–promoter interactions and to minimize the response time of the cell genetic apparatus to incoming stimuli. Pre-existing contacts were observed not only in differentiating cells, but also in mouse ESCs, where poised enhancers (marked with H3K4me1 and H3K27me3) contact with Polycomb-repressed genes to facilitate their expression upon differentiation [53,54]. A screening of the enhancers of 103 developmental genes in *Drosophila* showed that 94% of the enhancers have stable contacts with promoters before expression is activated during mesoderm development and that Pol II occurs in the paused state on the respective genes [55]. Thus, pre-existing contacts alone do not activate transcription, but they do prime the genes for transcription and allow the cell to immediately respond to stimuli. However, additional triggers are necessary for starting transcription; this role can be played by the recruitment of an activator, such as p53, TNF-alpha, or a tissue-specific developmental regulator. It should also be noted that a forced formation of loops between the enhancer and promoter may trigger gene transcription even in the absence of a key activator [56]. Pre-existing chromatin conformations might accelerate the creation of transcription factories or, alternatively, accelerate gene transfer to a transcription factory. This may be especially true for developmental genes expressed in narrow developmental windows.

Microscopic studies identified the threshold above which the bringing of a promoter and an enhancer together is not accompanied by gene activation. For example, the transcription of a reporter gene did not start when the gene–enhancer distance was reduced from 700 to 400 nm, but started when the distance was 330 nm in an insulator-pairing assay [57]. Similar results were obtained in a transvection assay across homologous chromosomes [18]. The enhancer–promoter approach was microscopically detected in a model of the ZRS (zone of polarizing activity regulatory sequence) enhancer and *Shh* promoter. The enhancer was spatially closer to the promoter in the limb bud part where *Shh* was active, and the distance between the elements was < 200 nm [58].

The other model is instructive and suggests that contacts form de novo in response to a stimulus or upon cell differentiation and that their formation is accompanied by simultaneous changes in gene expression [59,60,61,62,63,64]. 

Both of the two scenarios are simultaneously utilized during cell differentiation, which is accompanied by loss of pre-existing contacts, changes in chromatin state, and the acquisition of new contacts. A reorganization of contacts is a highly dynamic process that may take place within a few hours [16,65,66]. Thus, a short-term response to external stimuli is not associated with a global reorganization of enhancer–promoter contacts, but it is ensured by the 3D architecture of the genome and the total system of signal transmission from the cell membrane to the nucleus. While changes in cell fate in the course of cell specification are associated with a reorganization of enhancer–promoter contacts, the preformed chromatin topology facilitates a rapid progress of developmental programs in some cases—for example, in the case of developmental genes in *Drosophila*. 

As for the direct activation triggers, the role may be played not only by the distance between the enhancer and promoter, but also by their minor dynamic movements that occur within the preformed topology and are accompanied by the establishment of a limited number of new contacts [55,67]. The recruitment of a necessary transcription factor (TF) may also trigger activation, as was demonstrated, for example, for GATA3 and STAT6, which are recruited to the pre-existing conformation of the locus control region (LCR) and promoters of the IL-13, IL-4, and IL-5 genes in Th2 lymphocytes [68]. When transcription is activated in late erythroid cells, general transcription factors, Mediator, and Pol II are recruited not only to the promoter of the alpha globin gene, but also to distant regulatory elements. An analysis of certain mutants showed that assembly of the preinitiation complex on the enhancer is necessary for recruiting Pol II to the alpha globin gene promoter and that the process is accompanied by conformational changes in the elements (the promoter and enhancer are brought closer together) [69].

In sum, gene regulatory elements are highly mobile in the nuclear space. Molecular forces and specific chromatin proteins organize their movement, the formation of contacts of the elements, the assembly of transcription factories, as well as dissociation of the assemblies [39]. However, data from multiple gene-specific studies have still not been unified into a general concept to describe the molecular organization of enhancer-driven transcription in time and space.

## 4. Enhancers as Non-Coding RNA Transcription Units

### 4.1. Evidence of RNA Transcription from Enhancers

The discovery of mammalian enhancer transcription added another layer of complexity to our deepening understanding of the nature of these elements. New insights continue to upend long-held, common assumptions about enhancers, forcing us to re-examine the mechanisms of their action. The advent of the genomic era taught that the transcription of enhancers is a universal phenomenon characteristic of Metazoa [70,71,72,73,74]. In this chapter, we discuss the recent discoveries that shed light on the nature of this phenomenon and consider the possible role of enhancer transcription and enhancer RNA (eRNA) itself in regulating gene activity. Despite the presence of identical TFs and Pol II sites, promoters and enhancers were historically considered as two separate classes of regulatory elements [75]. Promoters are expected to be able to activate transcription at distinct sites, known as transcriptional start sites (TSSs). In turn, the enhancers are then able to activate transcription from the distal promoter, both remotely and regardless of orientation. It was believed that the enhancers themselves did not contain the sequences needed to initiate transcription. The first data on enhancer transcription was obtained in a well-characterized enhancer beta-globin gene cluster locus control region (LCR) [76,77]. This enhancer is 10–15 kb upstream of the gene cluster and comprises five DNAse I hypersensitivity sites (HS). Transcription initiation sites may be found across LCR, and eRNA transcription is shown to be tissue-specific and induced in response to transcription of the beta-globin genes [78]. Subsequent reports revealed a direct relationship between the tissue-specific transcription of eRNA and enhancer activity in mice and humans through genome-wide detection of RNA transcripts, using RNA-seq and Global Run-On sequencing (GRO-seq) [69,72,79,80,81]. A full 80% of the enhancers described in vivo exhibit tissue-specific transcription in mouse embryonic tissues, which correlates with enhancer activity [74]. The expression of genes targeted by the transcribing enhancer positively correlates with eRNA production and is considerably higher than the expression of genes targeted by the non-transcribing enhancers [82]. In line with these observations among mammals, *Drosophila* developmental enhancer transcription strongly correlates with the timing and location of enhancer activity [83]. 

Yet transcription is not found at all active enhancers. This suggests that eRNA is not required for the manifestation of enhancer activity, at least for this subset of active enhancers. Andersson et al. found that 20% to 33% of non-transcribed regulatory regions can activate transcription in an in vitro enhancer assay, suggesting that they may also function as enhancers without eRNA transcription [83,84,85].

### 4.2. Properties of eRNAs and Transcribing Enhancers

As promoters of protein-coding genes, enhancers expressing eRNA are enriched in transcription initiation complex, CBP/p300, and other TFs, and serine 5 phosphorylated RNA Pol II. Yet, unlike gene promoters, enhancers are depleted of serine 2 phosphorylated Pol II [86]. Promoters and enhancers are delimited by divergent transcription initiation sites and a well-positioned array of surrounding nucleosomes [87]. Transcribed enhancers are enriched in core promoter elements, including the initiator motif, leading to the proposal of a “unified architecture” between enhancer and promoter sequences, counter to earlier beliefs that they are distinctly separate [88]. However, promoters differ strongly from enhancers in their ability to be activated by enhancers: enhancer responsiveness [83,89]. It is thought that eRNAs are usually not spliced, do not leave the nucleus, are few in number, and are not often polyadenylated [71,72,84,90]. Polyadenylated eRNAs are rare and are typically synthesized from enhancers in one direction [71,75,91]. Bidirectionally transcribed enhancers are more common and produce non-polyadenylated transcripts [86,92].

A number of mammalian intragenic enhancers are transcribed to yield tissue-specific, long non-coding RNAs (lncRNA) in the direction of gene transcription [93]. Unlike eRNA, these RNAs are more stable and are spliced and polyadenylated. For mammals, it was shown that transcription in both directions from certain intragenic enhancers can attenuate the expression of the target gene by interfering with productive elongation [94]. The extent of the attenuation correlates positively with nascent eRNA expression. By analogy, it was demonstrated in *Drosophila* that IncRNA transcribed from the extragenic enhancers in the Bithorax complex (BX-C) can transcriptionally interfere with gene transcription [95]. These data indicate that long unidirectional eRNAs are different from short bidirectional unstable eRNAs and belong to a different class. eRNA transcription positively correlates with the enrichment of histone tags of active enhancers H3K27ac and the absence of repressive H3K27me3 tags [80,86]. However, recent findings suggest that the level of histone H3K4 methylation at enhancers corresponds to transcriptional activity so that highly active and highly transcribed enhancers display H3K4 trimethylation rather than H3K4 monomethylation, which is considered an intrinsic feature of all enhancers [96]. Active gene promoters typically exhibit trimethylated H3K4 (H3K4me3) [97]. Consequently, the difference in the H3K4 methylation patterns between enhancers and promoters simply reflects a different level of transcription from these elements.

### 4.3. Enhancers and Promoters Share Interchangeable Properties

The directionality of transcription appears to play a role in the ability to function as an enhancer or promoter in vivo. The relationship between eRNA transcription and enhancer activity during *Drosophila* embryogenesis was assessed in vivo in transgenic embryos [83]. Mikhaylichenko et al. demonstrated that the ability of enhancers to function as promoters in vivo strongly correlates with both the level of transcription and its directionality. However, this was not the case with the majority of elements (both enhancers and promoters) with unidirectional transcription [83]. The testing of gene promoters for enhancer activity revealed that promoters can function as enhancers in vivo when they are transcribed bidirectionally, while unididrectional promoters generally cannot. These proximal bidirectional elements appear to function as both an enhancer and a promoter for the same gene [83]. Evidence from *Drosophila* suggests that there is also a correlation between enhancer strength and endogenous Cap-analysis gene expression (CAGE) levels [98,99]. The stronger the promoter, the greater its ability to function as a housekeeping enhancer. In contrast, strong developmental enhancers are associated with a low level of transcriptional activity. This association of developmental enhancer strength with low endogenous expression levels indicates a possible regulatory trade-off between the promoter function and developmental enhancer function [99]. 

In recent years, massively parallel reporter assay (MPRA) and self-transcribing active regulatory region sequencing (STARR-seq) enabled quantitative and straightforward measurements of enhancer activity in mammals [87,100]. It was shown that gene promoters and distal regulatory regions have enhancer activity. By using STARR-seq on enriched targets, Lan T M Dao et al. found that a set of mammalian promoters display enhancer activity; the elements were named E-promoters [101]. A number of E-promoters were deleted using CRISPR/Cas9 system, and the results confirmed that they are involved in the *cis* regulation of distal genes in their natural context. A systematic analysis of the promoters of human coding genes revealed that up to 3% of promoters have enhancer activity and are involved in the regulation of distal genes through establishing long-range contacts [87,102]. Notably, enhancer-like promoters are generally associated with housekeeping and stress-response genes [101,103,104,105]. 

Taken together, these data indicate that transcriptional regulators have a wide range of activities. There are elements that function strictly as enhancers or promoters, while others work as enhancers with weak promoter activity, while some elements show both strong enhancer and promoter activity. This spectrum of activities tightly correlates with the directionality of their transcription, which likely reflects the underlying sequence properties of the element [83]. The above similarities between promoters and enhancers point to the unified architecture of transcriptional regulatory elements across Metazoa.

### 4.4. Pol II Pausing at Enhancers

Enhancer transcription proceeds through the same steps as gene promoter transcripts, including TSS-proximal pausing, capping, and release [98,106,107,108]. Early pausing was demonstrated for enhancer-like loci in human K562 cells and mouse embryonic fibroblasts (MEFs) [109]. Generally, early pausing is associated with high levels of uncapped transcripts and a smooth transition of uncapped to capped transcripts. Since early-paused transcripts only have 4–18 nt extending outside Pol II, it was speculated that short nascent eRNA and Pol II recruit DSIF and capping enzyme less efficiently than longer late-pause transcripts of coding genes [45,98,110]. Pol II at enhancers is particularly susceptible to early termination compared to promoters of coding genes. A high percentage of RNAs transcribed from enhancers were found to be oligo-adenylated at the 3′ end, which is a feature that facilitates degradation by the exosome [111]. The specific targeting of eRNAs to the exosome through oligo-adenylation is thought to play a central role in instituting the lack of stability that is a hallmark of eRNAs [98]. DSIF and NELF specify the correct post-transcriptional fate of small nuclear RNAs (snRNAs) through their association with Integrator, which is the large multisubunit complex responsible for the 3’ processing of pre-snRNAs [112,113]. Integrator is additionally implicated in transcription termination at enhancers [114]. Global nuclear run-on and Pol II profiling reveals a role of Integrator in the 3′-end cleavage of eRNA transcripts, leading to transcriptional termination. In the absence of Integrator, eRNAs remain bound to Pol II and their transcripts accumulate. Interestingly, the induction of eRNAs and gene expression responsiveness requires catalytic activity of the Integrator complex. These data point to a role of Integrator in both eRNA biogenesis and enhancer activation [114]. A recent study by Nathan D. Elrod et al. showed that Integrator can actively destabilize paused Pol II at the mRNA of coding genes as well. Similarly, Integrator utilizes its RNA endonuclease activity to cleave nascent RNA and drive the termination of paused Pol II [115]. Taken together, these findings reveal a previously unappreciated mechanism of transcription suppression inherent to promoters and enhancers alike. 

### 4.5. Functional Role of eRNA and Enhancer Transcription 

The functional role of eRNA remains largely unknown. We do know that the production of eRNAs is clearly associated with enhancer activity, which is observed in well-characterized enhancers (α-globin and β-globin loci) and globally in putative enhancers upon stimulation (as shown for p53, FoxA1, and estrogen receptor) [52,69,73,77,79,81].

How eRNAs contribute to transcriptional activation remains a subject of many ongoing investigations. There are three hypotheses regarding the role of enhancer transcription. The first states that the process of transcription, but not eRNA itself, is important for activating the capacity of enhancers. The second posits that eRNA transcripts are necessary for enhancer activity. The last suggests that eRNA can represent a transcriptional noise due to the promiscuous recruitment of Pol II to open chromatin [116]. The three hypotheses are not mutually exclusive, and there is evidence to support each of them [86,117]. Whether the process of enhancer transcription or eRNA itself plays the decisive role can vary from one locus to another [52,80,86,118]. Several recent lines of evidence support the functional role of some eRNA transcripts. The targeted degradation of eRNA using either RNA interference (RNAi) or locked nucleic acids was performed to test the functions of enhancer transcripts [108,117,119,120,121]. Specific eRNA knockdown was accompanied by the downregulation of coding genes, suggesting the functional importance for eRNAs [117]. While the features that constitute a functional eRNA are currently unknown, some studies implicated eRNAs in different steps of transcription regulation, including the modulation of chromatin accessibility at promoters and the release of the negative elongation factor NELF [71,122,123]. In particular, it was suggested that eRNAs mediate the looping interactions between enhancers and promoters of target genes and facilitate the recruitment of specific factors to stabilize the looping, such as cohesin and mediator [119,124,125,126]. However, treatment with flavopiridol to block eRNA productive elongation did not affect the enhancer–promoter looping at the P2RY2 and GREB1 loci following the estradiol stimulation of MCF-7 cells [79]. Furthermore, the inhibition of eRNA transcription affected neither enhancer-specific epigenetic modifications nor the recruitment of transcriptional regulators, suggesting that eRNA synthesis is not required for the assembly of enhancer–promoter complexes, at least in this context [79]. 

**Figure 3:**
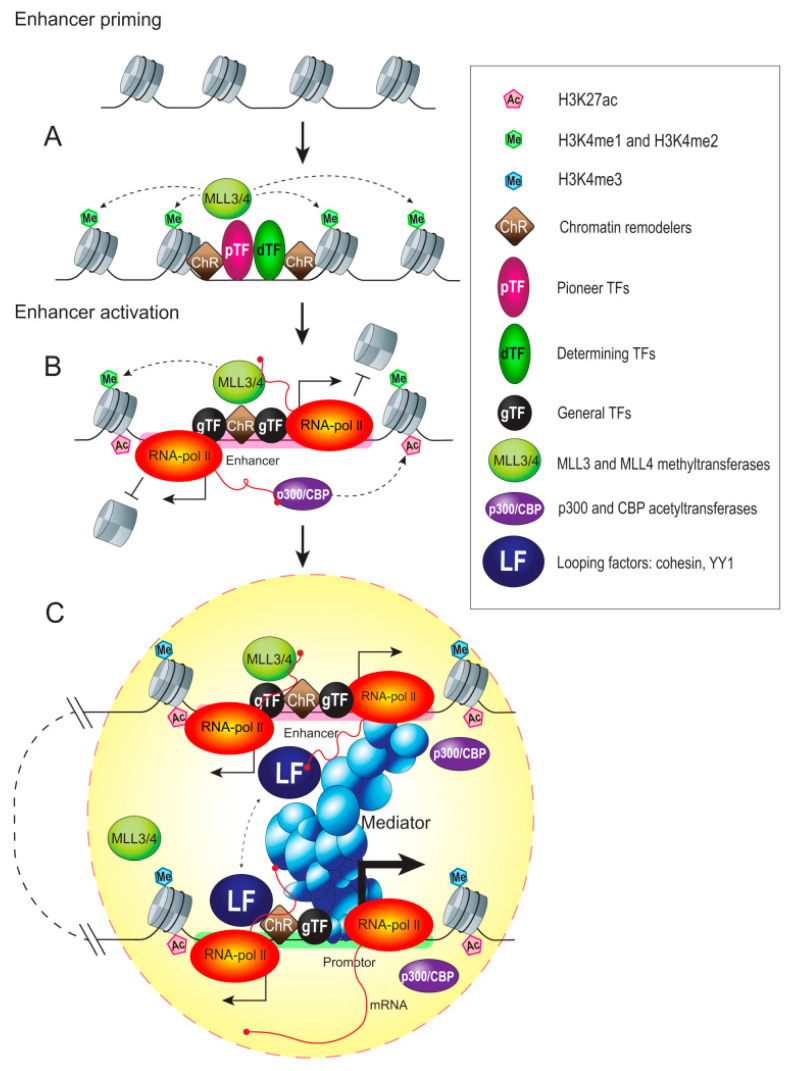
Activation of an enhancer and possible role of eRNA transcription in the process. The general diagram illustrates the stepwise activation of an enhancer as a transcription unit in response to developmental or other cues. (**A**) Assembly of transcriptional machinery at the enhancer is initiated by the binding of pioneer transcription factors (pTFs) [130]. Pioneer factors recognize their target DNA sequences in nucleosomes and trigger a remodeling of the adjoining chromatin landscape. This provides access to lineage-determining transcription factors (dTFs) to dictate the enhancer for activation in a specific cell type [131,132]. The enhancer is further primed by histone H3K4 mono-/di-methyltransferases, such as MLL3 and MLL4 [80]. In turn, H3K4me1 facilitates the recruitment of chromatin remodelers (ChR) to reposition the nucleosomes [133]. (**B**) The previous steps prepared the enhancer for the further recruitment of other cofactors, such as histone acetyltransferases. For example, CREB-binding protein and p300 (CBP/p300) are recruited to deposit histone acetylation marks and general transcription factors (GTFs) and Pol II, to initiate bidirectional transcription [117]. Transcription elongation could disrupt nucleosome occupancy [134] and evict nucleosomes locally, while Pol II pausing could protect the region from nucleosome reassembly [49], serving to maintain open chromatin. The C-terminal domain (CTD) of Pol II functions as a landing platform for a plethora of TFs and epigenetic regulators. The association of factors with the enhancer region is stimulated by their interactions with CTD and helps to create a local environment supportive for transcriptional activation. For example, histone-modifying enzymes interact with Pol II and deposit active chromatin marks during transcription elongation. p300 and CBP are associated with actively transcribing Pol II [135]. Hence, the act of transcription could serve to spread the H3K4me1/H3K4me2 and H3K27ac activating marks across the enhancer region [136]. H3K27ac recruits additional cofactors, such as bromodomain-containing protein 4 (BRD4), thus facilitating Pol II transition to elongation [80]. BRD4 is capable of directly interacting with eRNA [137]. This interaction strengthens the affinity of BRD4 for acetylated histones, enhances BRD4 and Pol II recruitment, and boosts transcription. (**C**) eRNA transcription and chromatin looping may occur concomitantly and precede gene transcription [138]. The recruitment of mediator and chromatin looping factors (LFs), such as cohesion and YY1, facilitates the enhancer–promoter interactions. eRNA can guide cohesin and YY1 localization and promoter–enhancer loop formation [139,140]. The presence of transcription machinery and RNA at enhancers provides multiple surfaces for stabilizing interactions with TFs and co-activators. eRNA may contribute to enhancer phase separation as a scaffolding, which is necessary for creating a high local concentration of factors (yellow bubble) favorable to transcriptional activity at promoters [141].

Additionally, RNA-tethering experiments in reporter assays demonstrated a quantitative effect of eRNA transcripts in conferring gene activation, independent of the act of transcription [108,117,119,127]. Contrary to these results, RNA exosome knockout cells, which have elevated steady-state eRNA levels without increased enhancer transcription, exhibit relatively unchanged nascent mRNA synthesis [128,129]. Several potential mechanisms whereby enhancer transcription and eRNA modulate the enhancer function are summarized in Figure 3. 

Experiments to discern the role of enhancer transcription or eRNAs are difficult to design. This is because alterations of the signals required for transcription may have negative, inadvertent effects on other aspects of enhancer activity. Recently, a different approach was used to manipulate eRNA transcription [142]. In this case, transcription was altered by depleting Spt5, which is a transcription factor that regulates Pol II pausing and processivity. A genome-wide analysis showed that approximately half of the examined enhancer–gene pairs exhibited correlated changes in transcription upon Spt5 depletion. In particular, Spt5 depletion led to loss of the super-enhancer–promoter physical interaction and gene expression at the immunoglobulin heavy-chain locus (Igh) [143] and consequently abrogated immunoglobulin class switching. This defect correlated strongly with a loss of enhancer transcription without affecting any other aspect of 3′RR enhancer activation, including the accessible chromatin state, the H3K27ac mark, the occupancy of Mediator, and cohesion. Nonetheless, the enhancer was unable to physically engage with its promoters. eRNA transcription was rescued by the CRISPRa-mediated recruitment of potent TFs to the 3′RR enhancers. Strikingly, the stimulation of transcription at one of the four 3′RR enhancers was sufficient to partly restore Ighg1 transcription in Spt5-depleted cells. To determine whether transcription is necessary for establishing and/or maintaining the steady-state interaction, transcription was inhibited in activated wild-type B cells with either triptolide, which abolishes transcription initiation [144], or flavopiridol, which inhibits P-TEFb-mediated release of paused Pol II, thus preventing elongation [145]. In both cases, 3′RR eRNA was significantly depleted. However, the enhancer–promoter interaction frequency remained the same or even slightly increased. This observation is consistent with previous data on the P2RY2 and GREB1 loci in MCF-7 cells [79]. Firz et al. concluded that in a subset of enhancers, transcription and/or Pol II is required for establishing the enhancer–promoter interaction after cell division or stimulation, but not for maintaining it. 

Most eRNAs function *in cis*, although there is growing evidence that they can exert the regulatory function in trans [122,140,146]. For example, MyoD enhancer regions activate transcription in part by giving rise to at least two eRNAs coherently regulating myogenesis [140,147]. The core enhancer eRNA (^CE^eRNA) regulates transcription of the adjacent MyoD gene in cis, whereas the distal regulatory region (DRR) enhancer transcript (^DRR^eRNA) promotes Myogenin transcription in trans [122,148,149]. Altogether, the data indicate that ^DRR^eRNA mediates loop formation through cohesin recruitment. A ^DRR^eRNA knockdown leads to reduced cohesin occupancy at the Myogenin locus, and this reduction downregulates Myogenin, ultimately preventing muscle cell differentiation. Furthermore, it was speculated that the process of cohesin recruitment mechanistically differs from estrogen receptor-regulated transcription, where the cohesin complex is present on many estrogen receptor-regulated enhancers even before ligand treatment and contributes to gene activation, at least in part, by stabilizing E2/ER-alpha/eRNA-induced enhancer–promoter looping [119,140]. As we discussed above, the majority of eRNAs are short-lived and not polyadenilated, which are the features limiting their range of action to neighbor genes [1]. By contrast, trans-acting ^DRR^eRNA, ncRNA-a7, KLK3eRNA, and Bloodlinc are polyadenylated [124,140,146]. Therefore, it is conceivable that polyadenylation may give eRNA stability to reach target genes on other chromosomes. 

A subset of other eRNAs was also found to interact with cohesin, which was proposed to facilitate communication between an enhancer and its target promoter by stabilizing looping [150]. However, recent studies indicate that the ablation of cohesin-mediated looping has only minor effects on the maintenance of gene expression on the genome scale [151,152]. The contacts that are lost without cohesin tend to be long range and represent topologically associated domain (TAD) boundaries. Shorter-range chromosomal contacts, especially those that connect active promoters with each other and with active enhancers, remain unaffected [153]. Mounting evidence point toward the existence of unknown cohesion-independent enhancer–promoter pairing mechanisms. Studies that implicate the eRNA function in cohesion recruitment should be considered with that caveat in mind. 

The TF Yin Yang 1 (YY1) was assumed to mediate enhancer loops. Unlike with cohesin, YY1 binding is strongly enriched at promoters and enhancers in mouse and human cell types [154,155]. Interestingly, a recent study demonstrates that eRNA binds YY1, which acts as a mediator of enhancer–promoter communication through dimerization. Reduced transcription from enhancers diminishes the YY1 occupancy at enhancers, whereas the artificial tethering of eRNA increases the YY1 occupancy [139]. 

All the aforementioned studies report average expression profiles, which have a limited potential to accurately describe what occurs in individual cells and at individual alleles. Fluorescence in situ hybridization (FISH) studies to measure eRNA and mRNA co-expression at individual alleles revealed that co-expressing alleles are rare, and co-expression rarely occurs in a closed enhancer–promoter configuration [55,85,156]. Rahman et al. speculate that the infrequent co-expression of eRNAs and mRNAs in a closed-loop configuration could indicate that eRNA transcription precedes looping or that eRNA transcription is mostly inhibited in the closed loop configuration [85]. Positioning of the nuclear receptor ERα, the co-activators, the mediator complex, and the basal transcription machinery in a closed loop configuration could indeed facilitate mRNA over eRNA transcription. Therefore, while the accumulation of eRNAs at active enhancers is unlikely to be required to stabilize the enhancer–promoter loops, eRNA transcription may nonetheless initiate this communication, which promotes target gene activation. Enhancer-mediated gene activation may be characterized by a feed-forward loop, whereby basal eRNA transcription facilitates the recruitment of TFs and co-regulators, which then further remodel chromatin and increase the frequency of eRNA and mRNA transcription initiation. Additionally, basal transcription at enhancers could maintain this regulatory region in an open chromatin state, thereby facilitating the binding of TFs and co-activators and allowing cells to respond rapidly to stimulation [33,80]. 

### 4.6. Conclusion

As presented in this chapter, the transcription of eRNAs may be more important for gene activation than was realized, with up to 50% of all enhancers depending on it for functioning [142]. Numerous functions are ascribed to eRNA transcription, including mediator and cohesin recruitment, the deposition of chromatin marks, enhancer–promoter looping, and phase separation, to name a few [119,124,141,157]. Available data can be contradictory. While some studies found a decisive correlation between eRNA transcription and the expression of neighboring genes [73,142], others suggest a lack of such correlation [158]. Yet mounting evidence outlined here points to a context-specific, rather than general, role of enhancer transcription in gene regulation. This notion could reconcile these conflicting reports.

As for the subset of enhancers that do not require eRNA transcription, nature has solved the problem of enhancer–promoter communication in a different way; exactly how is yet to be discovered. Some enhancers evolved to rely on transcription for functioning. For this other subset of enhancers, both the transcription process and some eRNAs by themselves are proven to have functional roles. More evidence is required to fully decipher the biological roles. To understand how eRNAs and TFs act—either jointly or independently—to stimulate transcription requires the targeted deletion of distinct enhancer regions to change the eRNA structure or abolish eRNA expression, while maintaining the binding of TFs. On the other hand, information on the dynamics of enhancer–promoter interactions in the presence or absence of eRNAs is still lacking and will require analyses of living cells at high spatial and temporal resolution. 

## 5. Epigenetic Chromatin Marks of Enhancers

### 5.1. Chromatin State of an Enhancer

One of the first epigenetic characteristics of enhancers is their depletion of nucleosomes as detectable by nuclease treatment. A significant correlation was revealed between cell-specific DNase I hypersensitive loci and enhancer elements [159]. In addition to the depletion itself, enhancer regions may carry hypermobile histone variant H3.3 and H2A.Z [160,161], which can be displaced from the DNA template easier than other variants. The enrichment in these histone variants is a characteristic feature of both active and poised enhancers and promoters [162]. Moreover, active enhancers are subject to nucleosome remodeling [163]. The following remodelers were found to function on enhancers: CHRAC and ACF [164], SWI/SNF [133,165], and CHD7 [166]. In sum, all these mechanisms promote the access of TFs to the DNA template [167].

The recruitment of multiple TFs is another characteristic feature of enhancers. Two modes of their binding to chromatin were proposed. The first is that the process is positively cooperative, which helps TFs overcome the nucleosomal barrier. The second is that there are certain pioneer factors that bind to nucleosomal DNA, thereby enabling other TFs to bind to the enhancer. The latter occurs via a direct effect on the nucleosome position, the recruitment of chromatin remodelers and modifiers, and the protection of DNA from methylation [130,168,169]. In addition to sequence-specific TFs, enhancers are characterized by the presence of the co-activators Mediator [170] and BRD4 [171] and the chromatin structural factors cohesin [172] and CTCF [173]. The Pol II elongation factor Ell3 marks enhancers in ES cells [174].

### 5.2. Histone Methylation

The nucleosomes that flank the TF binding sites in enhancers carry the specific histone marks H3K4me1 and H3K27ac [175,176,177,178]. The monomethylation of H3K4, the first mark of regulatory elements to be described, has a cell-specific distribution pattern in the genome [179]. However, this mark was also found in the 5′-upstream regions of active genes and has a broader distribution in the genome as compared with enhancers themselves. H3K4 monomethylation appears to be the first step, followed by nucleosome depletion, H3K27 acetylation, and enhancer activation (Figure 3). That is why H3K4me1 is important for enhancer priming [162,175,178]. This modification seems to mark both active and inactive enhancer regions [180,181]. However, not all potential enhancers in the genome are premarked with this modification, di- and trimethylation of H3K4 being found on enhancers as well [96,182].

The exact function of the H3K4me1 mark is still unclear. H3K4 methylation seems to affects the recruitment of some co-activators and co-repressors. There are several hypothetical players in this process. Methylated H3K4 can prevent the recruitment of some co-repressors, including DNA methyltransferase Dnmt3 [167]. Thus, H3K4me1 keeps the enhancer in a poised state by preventing repressive DNA methylation. Interestingly, preferential binding to H3K4me1 was described for acetyltransferase TIP60 [183], which participates in the loading of the aforementioned histone H2A.Z and its acetylation [184]. 

The methylation of H3K4 is accomplished by methyltransferases Trr1 in *Drosophila* and MLL3/4 in mammals [185,186,187]. In turn, MLL3/4 recruits the co-activator p300, which is necessary for establishing the mark of active enhancers (see below) [187,188]. The depletion of MLL3 and MLL4 results in the global loss of H3K4me1 and H3K27ac levels and Mediator and Pol II recruitment [185,186]. H3K4me1 is also important for enhancer–promoter contacts and facilitates the recruitment of the cohesin complex, which plays an important role in the formation of these contacts [189]. Finally, H3K4me1 facilitates the recruitment of the BAF remodeler, and nucleosomes carrying this modification are remodeled more efficiently [133]. 

At the same time, a broader range of methyltransferases may probably be involved in the formation of this modification. The mechanism of their recruitment is still unknown. It is likely that pioneer TFs are responsible for the recruitment to at least a certain group of enhancers [167]. Moreover, the H3K4me1 mark covers larger regions than TF binding sites do, which implies the spreading of activity from enhancers themselves to adjacent DNA regions. 

During enhancer disengagement, the methylation mark is erased by LSD1 demethylase in ESCs [190]. However, the H3K4me1 mark is detected on disengaged enhancers as well in *Drosophila* [181]. Another way to inactivate an enhancer is to establish H3K27me2 by the PRC2 complex, which blocks the acetylation of this residue [191].

In addition to H3K4me1, enhancers could carry H3K4me3 modification [96,98]. This mark is a classical feature of active promoters. However, as discussed above, recent studies indicated that promoter and enhancer activities could be intermingled, thus raising the concept of regulatory elements possessing different degrees of enhancer/promoter capability [192]. 

### 5.3. Histone Acetylation

Histone acetylation is a widespread modification of active chromatin. For the enhancer, there is an interplay of methylation and acetylation modifications: the characteristic H3K27ac mark is established on the enhancers that already carry H3K4me1 [181]. Different classes of TFs are able to recruit two paralogous histone acetyltransferases (HATs), p300 and CBP, onto enhancer chromatin [193]. Both HATs are regarded as specific factors for enhancers and are used to map the enhancers based on ChIP-seq data [167]. A greater part of p300 binding sites coincide with DNase I-sensitive sites [194]. P300/CBP acetylates multiple targets, including H3K18 and H3K27 residues. The presence of the latter mark distinguishes active enhancers from poised ones [167]. However, a group of enhancers carries p300 while lacking H3K27ac in human ESCs [162]. Thus, acetylation on enhancers is under strict control, the details of which are as yet unknown.

However, not all enhancers carry a p300/CBP marker, and some other HATs appear to bind to them. For example, Gcn5/PCAF HAT and its preferential H3K9ac mark were detected on enhancers [178,195,196]. It seems that an enhancer may carry a number of HATs, whose repertoire depends on the spectrum of TFs bound to the given enhancer. Moreover, H3K27ac is not an obligatory mark of active enhancers [181,197]: there are enhancers that are devoid of the enhancer-specific chromatin marks described above [198].

Acetylated histones are recognized by bromodomains, which are found in various proteins (including general TFs, chromatin modifiers, and remodelers). The acetylated histone–bromodomain interaction can stabilize the protein assemblies on enhancers. Acetylation can also destabilize nucleosomes and contribute to the formation of histone-free chromatin regions [199]. Moreover, histone tails were shown to participate in long-range interactions in nucleosomal arrays [200], suggesting that their acetylation may contribute to this important process [167].

There is another class of enhancers, referred to as poised enhancers, which were described in ESCs [162,178]. Such enhancers are located close to developmental genes; they are lineage-specific and are activated during differentiation. They contain the H3K4me1 mark but lack the H3K27ac mark; they also contain the H3K27me3 mark and are occupied by the polycomb complex PRC2. Poised enhancers start to drive transcription only during cell differentiation, simultaneously with H3K27me3 substitution by H3K27ac. Thus, poised and active enhancers are distinguished by trimethylation or acetylation of the H3K27 residue, while both groups carry the H3K4me1 mark [162,175]. In *Drosophila*, H3K27me3 histone demethylase UTX directly binds CBP/p300, which helps to change H3K27me3 into H3K27ac and activate poised enhancers [201].

It is as yet unclear whether the H3K4me1 or H3K27ac modifications are sufficient or necessary for enhancer activity. Enhancers show heterogeneous histone modifications and, in addition to the aforementioned marks, they may carry other ones, such as H3K9ac and H3K18ac [178,202], H3K79me2/3 [181,203], H3K9me3 [178], H3S10ph and H3S28ph [204], and lysine crotonylation [205]. Interestingly, histone modifications can determine (or reflect) the enhancer orientation; i.e., asymmetry was described in histone modification and nucleosome positioning relative to a TF binding site [206]. 

### 5.4. DNA Modification

Finally, the epigenetic mark of DNA methylation was found on inactive enhancers [207,208,209,210,211]. In general, there is an anticorrelation in the recruitment of TFs and co-activators and DNA methylation. This is in line with current views on DNA methylation as a marker of gene silencing. Another DNA modification, 5-hydroxymethylcytosine (5hmC), shows the opposite behavior [212,213,214]. The modification positively correlates with gene activity, may be found on both active and poised enhancers [215], and coincides with the H3K4me1 and H3K27ac marks [169,214]. Interestingly, 5hmC flanks the TF binding site, while 5-methylcytosine (5mC) marks the TF binding site itself [215].

In sum, enhancers are dynamic entities that carry different epigenetics marks at different developmental stages. Epigenetic marks can participate in the assembly of transcription complexes, open access for TF binding, and prime regulatory elements [167]. However, the mechanisms of these processes have yet to be studied in detail.

## 6. Perspectives

Recent advances in the techniques available to study the molecular processes in the nucleus contributed a lot into our understanding of enhancer action. At the same time, novel models brought novel issues, and several of them should be mentioned. The precise molecular mechanism that various enhancers use to control the transcription burst duration and frequency is still unknown. The molecular forces that drive the formation of sub-nuclear bodies, including transcription factories, are under intense study now. The contributions of different classes of chromatin-associated molecules, including cohesins and eRNAs, to the formation of enhancer–promoter contacts need thorough investigation. Different molecular landscapes and potentially different modes of action of different classes of enhancers and promoters require substantial attention. Finally, the arising concept of a mixing of different types of activities, such as enhancer, promoter, and insulator activities, in one regulatory element is attracting a lot of interest.
